# From Child to Old Man: A Slowly Evolving Case of Chromoblastomycosis Caused by *Cladosporium cladosporioides*

**DOI:** 10.3390/antibiotics12121713

**Published:** 2023-12-09

**Authors:** Carmen Rodríguez-Cerdeira, Rigoberto Hernández-Castro, Roberto Arenas, Cecilia Sandoval-Tress, Fidencio Gutiérrez-Murillo, Luary Carolina Martínez-Chavarría, Juan Xicohtencatl-Cortes, Monika Fida, Erick Martinez-Herrera

**Affiliations:** 1 Fundación Vithas, Grupo Hospitalario Vithas, 28043 Madrid, Spain; rarenas97@hotmail.com; 2 Dermatology Department, Hospital do Vithas, 36206 Vigo, Spain; 3 European Women’s Dermatologic and Venereologic Society, 36700 Tui, Spain; monikafida@gmail.com; 4 Psychodermatology Task Force of the Ibero-Latin American College of Dermatology (CILAD), Buenos Aires C1091, Argentina; 5 Departamento de Ecología y Agentes Patógenos, Hospital General Dr. Manuel Gea González, Tlalpan 14080, Mexico; rigo37@gmail.com; 6 Sección de Micología, Hospital General “Dr. Manuel Gea González”, Tlalpan 14080, Mexico; 7 Departamento de Dermatología, Hospital General de Zona # 42 Instituto Mexicano del Seguro Social, Puerto Vallarta 48310, Mexico; cecytress@hotmail.com; 8 Dermatólogo Practica Privada, Reynosa 87390, Mexico; mydermatologyclinic@gmail.com; 9 Departamento de Patología, Facultad de Medicina Veterinaria y Zootecnia, Universidad Nacional Autónoma de México, Coyoacán 04510, Mexico; luary@unam.mx; 10 Laboratorio de Bacteriología Intestinal, Hospital Infantil de México Dr. Federico Gómez, Cuauhtémoc 06720, Mexico; juanxico@yahoo.com; 11 Dermatology Department, Medical University of Tirana, U.M.T., 1001 Tirana, Albania; 12 Sección de Estudios de Posgrado e Investigación, Escuela Superior de Medicina, Instituto Politécnico Nacional, Plan de San Luis y Díaz Mirón, Ciudad de México 11340, Mexico

**Keywords:** chromoblastomycosis, *Cladosporium cladosporioides*, ITS1-2 rRNA region, *TEF-1a* gene

## Abstract

Chromoblastomycosis is a chronic granulomatous mycosis of the skin and subcutaneous tissue caused by traumatic inoculation with dematiaceous fungi. This disease primarily affects agricultural workers, who are mostly men. We present a case of chromoblastomycosis in a 63-year-old male farmer patient with dermatosis over 50 years of evolution, with warty, erythematous, and scaly plaques that predominate on the left hemithorax. Direct examination with potassium hydroxide (KOH) revealed numerous fumagoid cells. Amplification and sequencing of the internal transcribed spacer (ITS) and translation elongation factor 1-alpha *(TEF-1a)* gene revealed that chromoblastomycosis was caused by *Cladosporium cladosporioides*. The chromoblastomycosis was treated with itraconazole and fluconazole without any improvement, and amphotericin B was administered with partial improvement.

## 1. Introduction

Chromoblastomycosis is a chronic subcutaneous mycosis endemic to tropical and subtropical regions. It generally affects people in poor regions of Africa, Asia, South America, Central America, and the Caribbean. However, there are reports of its incidence in some countries in Europe, Canada, Russia, and Japan. It is caused by melanised fungi that are generally found in soil or plants and is classified as an orphan neglected disease [1,2,3]. The route of infection is frequently through a wound or lesion in the skin. The most common sites are the feet, knees, lower legs, and hands. However, it has also been reported in other sites or areas, such as the nose, auricular region, cornea, conjunctiva, scapular region, axillae, abdomen, buttocks, and as a phagedenic ulcer on the face [2,3].

The presence of muriform cells in the microscopic diagnosis of clinical samples allows for the rapid determination of sufficient evidence of the presence of the dematiaceous fungi responsible for chromoblastomycosis. Nevertheless, the clinical characteristics and therapeutic response may vary between patients due to the different etiological agents involved. Therefore, identification at the species or at least at the genus level is essential to choose the most appropriate antifungal treatment [2,4].

The most frequent etiological agents belong to the genera *Fonsecaea* and *Cladophialophora*; however, there are also case reports of *Chaetomium*, *Cyphellophora*, *Exophiala*, *Phialophora*, *Cladosporium*, *Phoma*, and *Rhinocladiella* [2,4]. Members of the genus *Cladosporium* (*Cladosporiaceae*, *Capnodiales*) can be isolated from almost any environmental source and geographic location, including soil, plants, and manure, and they represent the most common isolated airborne fungi [5,6]. The genus is characterised by the distinctive form of its conidiophores, which are erect, straight, or geniculate, proliferating mostly sympodially and forming unbranched or branched acropetal conidial chains of smooth to roughened dry conidia. However, the most characteristic feature is the presence of a thick refractive to darkened cladosporioid or coronate scar, defined as a raised periclinal rim with a central convex dome [2,7].

Within the group of species that affect humans, *C*. *bantiana*, *C*. *carrionii*, and *C*. *devriesii*, characterised by the absence of conidiophores and unpigmented conidial scars, were reclassified in the *Cladophialophora* genus using molecular studies. This action generated a reduction in the number of species of medical interest. Through morphological datasets and molecular studies using 18S-ITS1-5.8S-ITS2-28S rRNA, actin, and translation elongation factor 1-α gene sequences, more than 169 species have been distinguished. It has also been shown that *C. cladosporioides*, C. herbarum, and *C. sphaerospermum* are species complexes encompassing several sibling species that can only be differentiated by molecular techniques [5,8,9].

Among the species most frequently associated with infections in humans are *C*. *oxysporum* (cutaneous phaeohyphomycosis with morphological identification), *C*. *sphaerospermum* (subcutaneous infection with ITS1-ITS4 molecular identification), *C*. *herbarum* (chronic rhinosinusitis with 18S rRNA molecular identification), *C*. *macrocarpum* (brain abscess with morphological identification), *C*. *tenuissimum* (chromoblastomycosis with ITS and D1/D2 and a D1/D2 domain of 26S rRNA molecular identification), and *C*. *langeronii* (chromoblastomycosis with ITS1-ITS4 molecular identification) [10,11,12,13,14]. Reports of *C. cladosporioides* infection in humans include cases of phaeohyphomycosis identified by morphological identification, keratomycosis with ITS1-18S rRNA molecular identification, pneumonia with morphological identification, rhinosinusitis with morphological identification, and, rarely, chromoblastomycosis with a D1/D2 domain of 26S rRNA molecular identification [15,16,17,18,19]. However, in most cases, morphological identification was used to identify the etiological agent, or the molecular marker was not able to differentiate the subspecies within the *C*. *cladosporioides* complex; therefore, specific identification in many cases is incomplete.

In the present study, we report a slowly evolving clinical case of chromoblastomycosis caused by *C. cladosporioides* in a Mexican farmer.

## 2. Case

**Figure 1:**
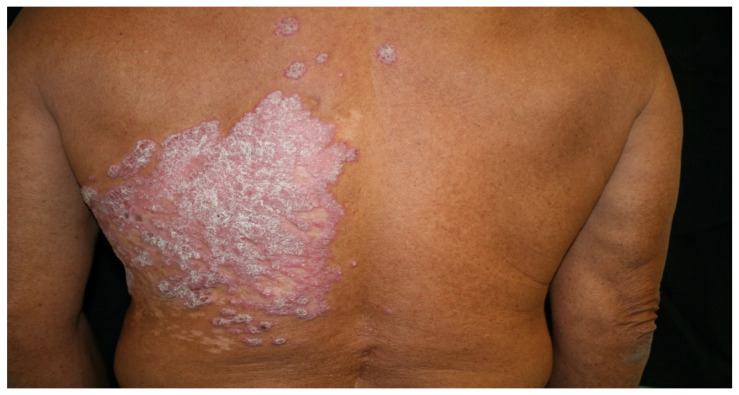
Verrucous plaques surrounded by a few nodules and erosions on the back of a patient with chromoblastomycosis.

We present the case of a 63-year-old male farmer working in an area with a warm climate, who was indigenous and a resident of Nayarit State, Mexico. He visited the clinic for localised, asymmetrical dermatosis affecting the left hemithorax, consisting of a warty, nodular, erythematous, scaly plaque measuring 21 × 30 cm in diameter, with a rough surface, well-defined edges, and chronic evolution with multiple satellite lesions (Figure 1).

**Figure 2:**
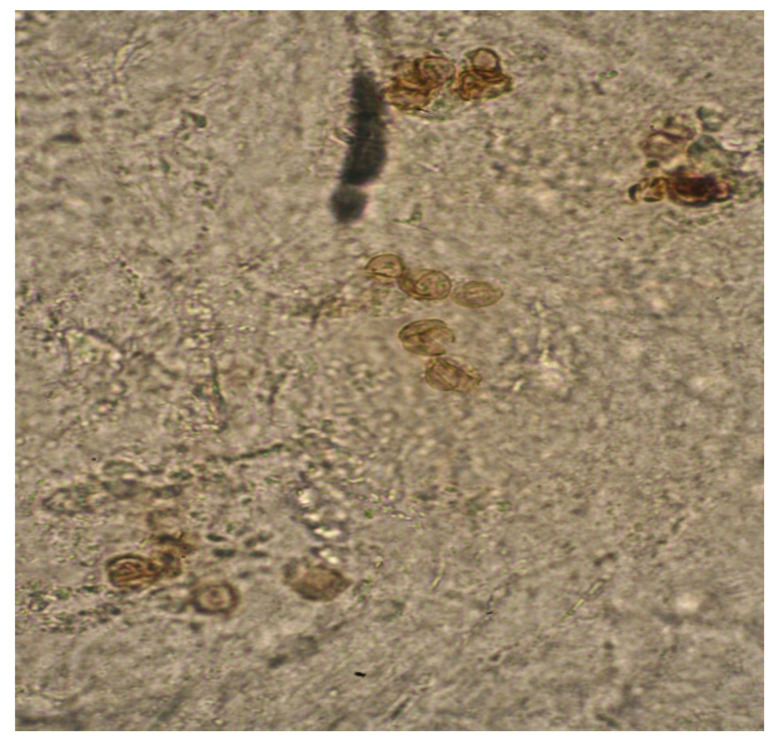
Direct examination showing clumped and septate fumagoid cells (×400).

**Figure 3:**
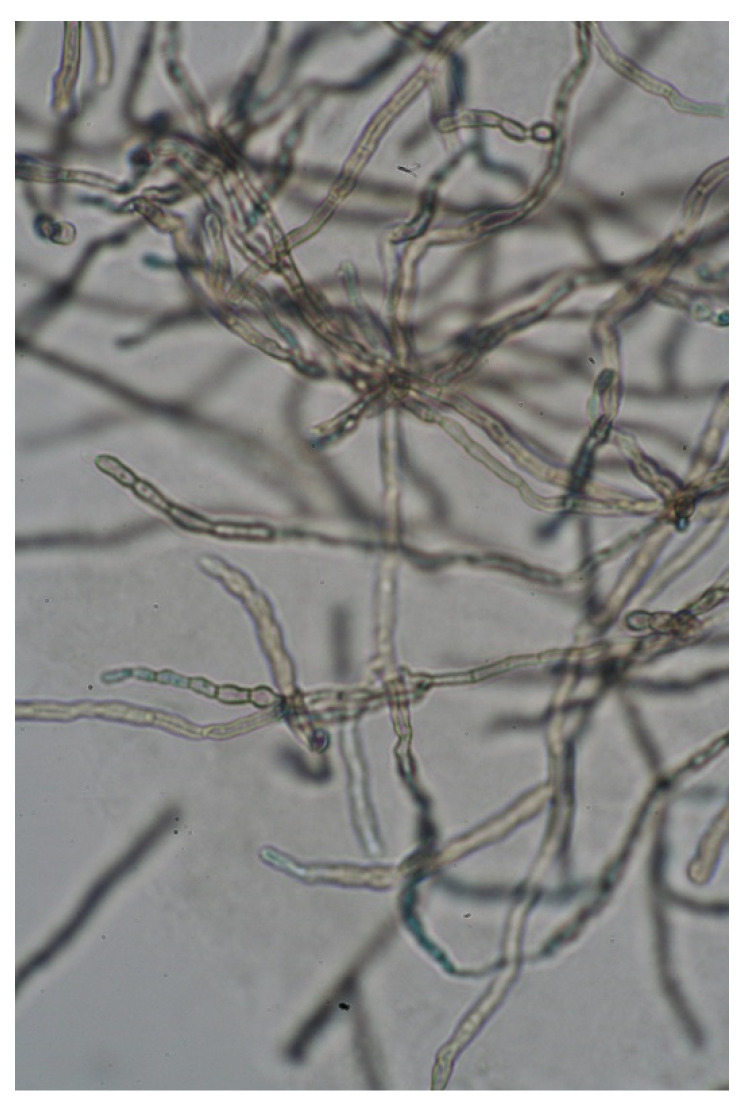
Septate hyphae and elliptic conidia of *C. cladosporioides* (×400).

The onset of the lesion was at 10 years of age after trauma to the left hemithorax and was characterised as being asymptomatic and progressively growing. Direct examination of the scales with 10% potassium hydroxide (KOH) showed abundant fumagoid cells (Figure 2), and a culture was performed on Sabouraud agar with 10 days of incubation at 26 °C. After 10 days, black colonies with a cottony texture and grey shading were observed; the conidia were microscopically observed as forming chains (Figure 3).

Molecular identification was performed by PCR amplification and sequencing of the ribosomal region 18S-internal transcribed spacer (ITS)1-5.8S-ITS2-28S rRNA and the translation elongation factor 1-alpha (*TEF-1a*) genes. DNA was extracted from biopsies of skin lesions and fungal isolation using a commercial DNeasy blood and tissue system (Qiagen, La Jolla, CA, USA), according to the manufacturer’s instructions.

For the amplification of 18S-ITS1-5.8S-ITS2-28S rRNA and translation elongation factor 1-alpha (*TEF-1a*), the previously described primers were used [20,21,22]. In the PCR of the skin lesion, two amplification products were observed: one at 550 bp and another at 650 bp. The products were purified using a QIAquick PCR Purification Kit (Qiagen, La Jolla, CA, USA) according to the manufacturer’s instructions. Then, the products were sequenced in both directions using the same primers with Taq FS Dye Terminator Cycle Sequencing fluorescence-based sequencing and analysed on an Applied Biosystems 3730 DNA sequencing system (Foster City, CA, USA). The subsequent nucleotide sequences of both fragments were edited using a Vector NTI advance 11.5 and compared with the GenBank database to determine their homology using the BLAST nucleotide program of the National Centre of Biotechnology Information (NCBI). The 550 bp product exhibited 100% homology with members of the *C. cladosporioides* complex (*C. cladosporioides*, *C. pseudocladosporioides*, *C*. *angustisporum*, *C*. *colombiae*, *C*. *delicatum*, *C*. *europaeum*, *C*. *inversicolor*, and *C*. *vicinum*, among others), and the 650 bp amplification product exhibited 100% homology with members of the *Candida parapsilosis* complex. As the final identification was not defined, the *TEF-1a* gene was amplified from the DNA extracted from the biopsy. Only a 400 bp product was amplified, purified, and sequenced in both directions. 

The *TEF-1a* sequence exhibited 100% homology with *C*. *cladosporioides* HM148291, MZ344181, and ON409522, among others. No amplification product was observed for the *C. parapsilosis* complex. Additionally, DNA was extracted from the culture of the isolated fungus, and the 550 bp and 400 bp products (18S-ITS1-5.8S-ITS2-28S rRNA and *TEF-1a*, respectively) were amplified. The sequences showed the same results as the skin biopsy products. The sequences were deposited in GenBank under accession numbers OQ851636 (*C. cladosporioides* complex 18S-ITS1-5.8S-ITS2-28S rRNA), OQ857391 (*C. parapsilosis* complex 18S-ITS1-5.8S-ITS2-28S rRNA), and OQ865375 (C. *cladosporioides TEF-1a*).

Multiple alignments were established using all the sequences of the Cladosporium species available in GenBank with Clustal W algorithms included in MEGA software version 7.0.26 [20]. Phylogenetic reconstruction was conducted using a Bayesian approach with MrBayes version 3.2 [21]. The analysis was performed for 2,000,000 generations with sampling trees every 100 generations.

**Figure 4:**
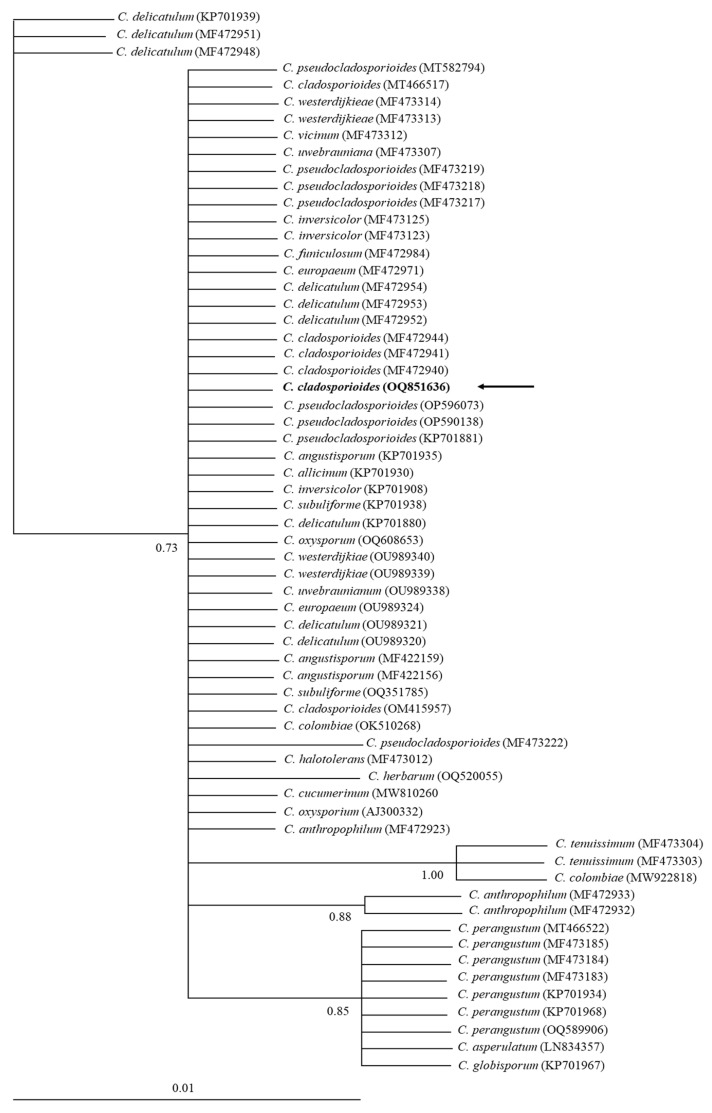
Bayesian tree, inferred by distance-based analysis of 18S-ITS1-5.8S-ITS2-28S rRNA sequence of *C. cladosporioides* complex and other related *Cladosporium* species. The numbers of the nodes indicate the values of support or posterior probability. GenBank accession numbers are shown at the end of each branch. The samples obtained in this study are indicated in bold and with arrows.

**Figure 5:**
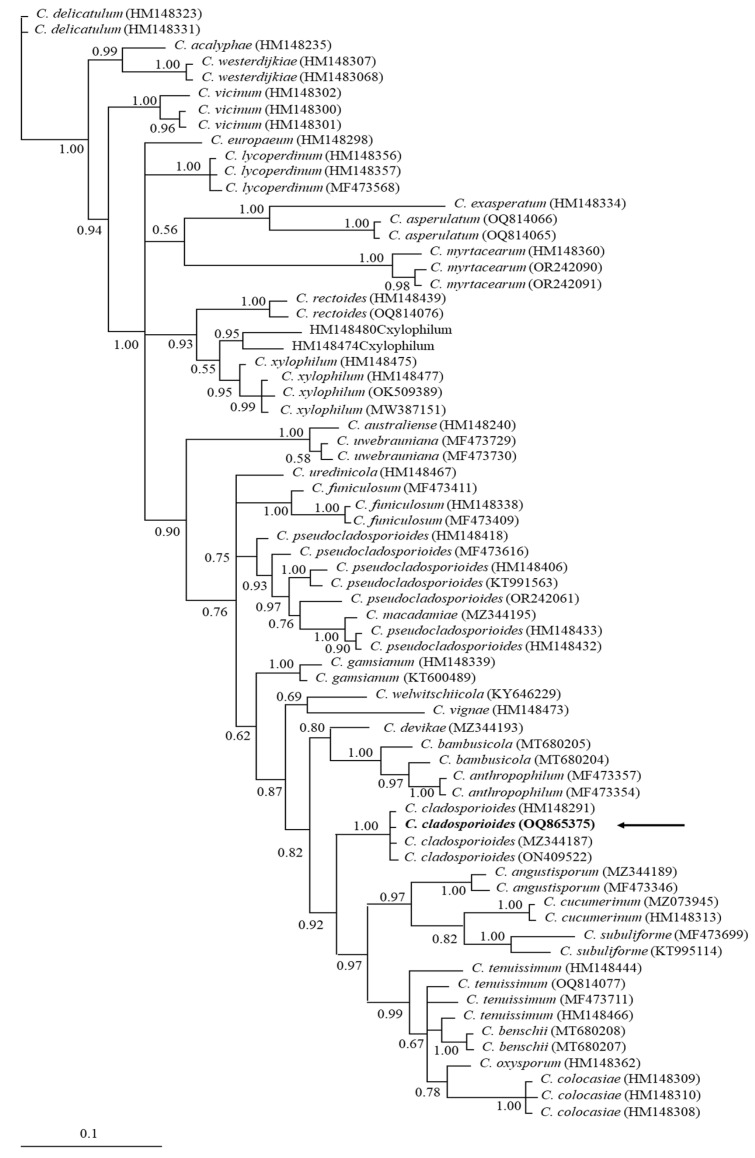
Bayesian tree, inferred by distance-based analysis of translation elongation factor 1-alpha (*tef-1*) partial sequence of *C. cladosporioides* and other related *Cladosporium* species. The numbers of the nodes indicate the values of support or posterior probability. GenBank accession numbers are shown at the end of each branch. The sample obtained in this study is indicated in bold and with an arrow.

The phylogenetic tree with the 18S-ITS1-5.8S-ITS2-28S rRNA sequences showed polytomies among several *Cladosporium* species forming a *C. cladosporioides* complex (Figure 4). This is in contrast with *TEF-1a*, whose sequence supported the grouping with *C. cladosporioides* with high posterior probability values (1.00) (Figure 5).

This study was approved by the Institutional Review Board of Hospital General “Dr. Manuel Gea Gonzalez”, Mexico City, Mexico (No. 06-58-2016) [22,23].

**Figure 6:**
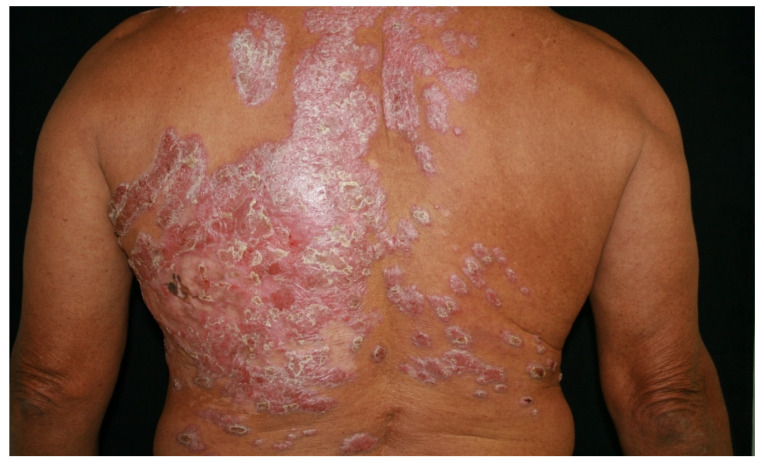
Healed lesions of chromoblastomycosis following treatment.

The patient was initially treated with itraconazole (200 mg/d) for 5 years. However, the antifungal agent was ineffective, and treatment was changed to fluconazole for 3 years, starting at a dose of 200 mg/d until reaching a dose of 400 mg/d. The patient did not respond to treatment, and the lesions began to spread, affecting the right hemithorax (Figure 6).

Therefore, the treatment was changed to amphotericin B (1 mg/kg of body weight), resulting in partial improvement, but with persistent lesions.

The patient developed hypertension due to adverse effects of the drug, and treatment was discontinued. Currently, he is being treated again with fluconazole at a dose of 200 mg/d, topical keratolytics (semi-occlusive cold cream (once daily)), and 30% urea cream (twice daily) for persistent skin lesions.

## 3. Discussion

Chromoblastomycosis is a chronic, subcutaneous, granulomatous, and progressive skin infection caused by different species of fungi belonging to the *Herpotrichiellaceae* family. The disease is considered an occupational disease, occurring among individuals working in agriculture such as farmers, gardeners, lumberjacks, and agricultural product traders, particularly in low-income countries [6,24,25]. *C. cladosporioides* has been associated with haemorrhagic pneumonia and cutaneous diseases such as phaeohyphomycosis and mycetoma, and, rarely, with chromoblastomycosis [17,19,26,27,28]. The infection begins with the inoculation of dematiaceous fungi from an environmental source through a skin lesion. The chromoblastomycosis is characterised by presentation in different clinical forms; the polymorphism of lesions can include the presence of plaques, nodular lesions, verrucous or cauliflower-like lesions, tumours, and cicatricial or atrophic lesions [2].

The diagnosis of chromoblastomycosis includes conventional methods, such as direct, histopathological, and fungal isolation methods. Direct microscopy and histopathology can also be performed with the primary objective of visualising muriform cells, which are pathognomonic of chromoblastomycosis.

Histopathologically, chromoblastomycosis is characterised by hyper-parakeratosis, pseudoepitheliomatous epidermal hyperplasia, microabscesses, pyogranulomatous reactions, and irregular acanthosis alternating with areas of atrophy. The dermis usually presents dense granulomatous inflammation with different grades of fibrosis, associated with mononuclear cells (histiocytes, lymphocytes, and plasma cells), epithelioid cells, giant cells, and polymorphonuclear cells [2,3].

Fungal isolation is generally performed on Sabouraud dextrose agar; the identification of the fungi is performed by observing characteristic, dark-pigmented colonies after 3 weeks of culture. However, these methods are time-consuming and have poor sensitivity and specificity. Therefore, molecular analysis is required for a rapid and more specific identification of the appropriate molecular marker to facilitate correct identification in terms of the genus and species involved, especially when it comes to mixed infections, as was the case in this instance. The 18S-ITS1-5.8S-ITS2-28S ribosomal region has been widely used as a marker for identification in cases of chromoblastomycosis and is considered the best molecular marker to identify this type of fungal agent [2,3].

However, when the 18S-ITS1-5.8S-ITS2-28S ribosomal region is not able to define the species or subspecies within a particular genus, other molecular markers can be used, such as translation elongation factor 1-α, β-tubulin, RNA polymerase II, and calmodulin. It is also recommended to perform a multilocus analysis to differentiate subspecies or sibling species. The genes used for the analysis are the ITS region, β-tubulin (BT2), elongation factor 1α (*TEF-1a*), actin (ACT1), and cell division cycle42 (Cdc42) genes [29].

We used the ribosomal region as the first step in molecular identification. The 18S-ITS1-5.8S-ITS2-28S genetic marker allowed us to identify a *C. cladosporioides* complex. Since we did not define the species using the ITS-rRNA region, we decided to amplify and sequence the *TEF-1a* gene [30]. Sequence analysis allowed us to identify *C. cladosporioides* as the causal agent. Another alternative for rapid and precise molecular identification is the matrix-assisted laser desorption ionisation–time of flight (MALDI-TOF) technique. This identification method has been shown to contain a cost-effective robust commercial database and allows for specific identification at the species level of an extensive variety of fungi and yeasts [31].

In the patient’s skin sample, we identified *C. cladosporioides* and the *C. parapsilosis* complex (*C. parapsilosis* sensu lato, also known as the *C. parapsilosis* complex, which includes *C. parapsilosis* as the most frequent medical pathogen, followed by *C. orthopsilosis* and *C. metapsilosis*, formerly known as *C. parapsilosis* Group I, II, and III, respectively).

The *C*. *parapsilosis* complex has been frequently misidentified using conventional biochemical tests, leading to an underestimation of the frequency of *C*. *orthopsilosis* and *C*. *metapsilosis*. Molecular identification is the best option to differentiate the three species of the complex. However, the introduction of MALDI-TOF has become a rapid and reliable technique for identifying cryptic species in the clinical setting.

The *C. parapsilosis* complex is a normal inhabitant of the skin, nails, and diverse mucosal areas of humans and mammals. It is a common cause of opportunistic infections and is related with both high morbidity and mortality rates in hospitalised immuno-compromised patients. It has also been linked to systemic bloodstream infections in intensive care units (ICUs) worldwide [32]. Recently, *C*. *parapsilosis* infections have been observed associated with a strong increase in resistance to azoles, including various nosocomial (hospital-associated) outbreaks associated with resistance to fluconazole [33,34,35,36].

It has been reported that patients with chromoblastomycosis who are diagnosed after several years with clinical manifestations as well as places where antifungal treatment is not available or is economically inaccessible are factors that can increase the risk of sequelae, such as bacterial infections or co-infections with yeast or fungi [3,37,38].

In our case, we could not determine the role of the *C*. *parapsilosis* complex within this extended clinical case of chromoblastomycosis. With this finding, we were not able to determine whether the presence of the *C*. *parapsilosis* complex had had an important effect on the course of the infection, as well as an impact on treatment. The main problem was that we were not able to isolate *C*. *parapsilosis* from the clinical sample, as the identification through amplification and sequencing of the 18S-ITS1-5.8S-ITS2-28S rRNA region only allowed the identification of the *C*. *parapsilosis* complex. In addition, when we attempted to amplify genetic markers that would allow for the identification of the *C. parapsilosis* complex, we did not obtain conclusive results.

In general, the treatment of chromoblastomycosis is complicated and challenging for clinicians. It is generally associated with low cure rates and high relapse rates, especially in chronic and extensive cases. To determine the most appropriate treatment for a patient, the causative agent, extent of the condition, topography, and general state of the patient’s health must be considered. The treatment consists of long periods of antifungal therapy combined with physical treatments, such as surgery, cryotherapy, laser therapy, and photodynamic therapy [15,32].

Surgery is the best physical method for the treatment of chromoblastomycosis, especially in small and localised lesions, where excisional surgery is recommended for all initial small and well-delimited cutaneous lesions. Surgery can be used in combination with itraconazole or terbinafine antifungal treatment. Cryotherapy with liquid nitrogen and thermotherapy demonstrate minimal risk of adverse effects, and these physical treatment options are relatively accessible, but are more recommended for limited and small lesions [2,3].

There are multiple therapeutic options, and the following antifungal agents can be used: 5-fluorocytosine, 5-fluorouracil, thiabendazole, amphotericin B, ketoconazole, fluconazole, itraconazole, terbinafine, and posaconazole.

The most efficacious antifungal agents are itraconazole (200–400 mg/day) and terbinafine (500–1000 mg/day) over a period of 6–12 months. The combinations of itraconazole/cryosurgery, terbinafine/cryosurgery, itraconazole/terbinafine, itraconazole, and/or terbinafine with local heat application are the best treatment alternatives [39,40,41]. The combination of itraconazole and terbinafine fungal therapy is often used in patients with refractory forms. In recent years, other agents, such as posaconazole, an expanded spectrum triazole, have become the best potential alternatives for the treatment of all clinical forms of chromoblastomycosis, including severe or refractory clinical presentations [42].

The patient was initially treated with itraconazole and fluconazole without favourable results; therefore, we decided to use amphotericin B, which exhibited partial favourable results. The use of keratolytics (urea 30%) with fluconazole is currently prescribed as maintenance therapy for this patient.

## 4. Conclusions

In conclusion, a clinical case of chromoblastomycosis in a farmer working in an area with a warm climate in western Mexico was described. Correct and timely clinical diagnosis is essential because chromoblastomycosis lesions are clinically polymorphic and commonly misdiagnosed. In this case, the causative agent (*C. cladosporioides*) was identified using molecular biological techniques in a patient who had lived with the disease for more than 50 years. If not diagnosed at an early stage, the disease may be refractory to antifungal therapy. Itraconazole is the most effective drug for chromoblastomycosis; however, it was ineffective in our patient. When this drug is replaced with amphotericin B, complementary therapies such as keratolytics can be effective. Currently, the patient has maintained an acceptable response to a combination of fluconazole and cold cream with 30% urea.

## Data Availability

Data are contained within the article.

## References

[1] Guevara A., Nery A.F., de Souza Carvalho M.M., Bonfietti L., Rodrigues A.M., Hagen F., de Carvalho J.A., de Camargo Z.P., de Souza Lima B.J.F., Vicente V.A. (2022). Molecular epidemiology and clinical-laboratory aspects of chromoblastomycosis in Mato Grosso, Brazil. Mycoses.

[2] de Brito A.C., Bittencourt M.J.S. (2018). Chromoblastomycosis: An etiological, epidemiological, clinical, diagnostic, and treatment update. An. Bras. Dermatol..

[3] Queiroz-Telles F., de Hoog S., Santos D.W., Salgado C.G., Vicente V.A., Bonifaz A., Roilides E., Xi L., de Maria Pedrozo E Silva Azevedo C., da Silva M.B. (2017). Chromoblastomycosis. Clin. Microbiol. Rev..

[4] López Martínez R., Méndez Tovar L.J. (2007). Chromoblastomycosis. Clin. Dermatol..

[5] Becchimanzi A., Zimowska B., Nicoletti R. (2021). Cryptic diversity in *Cladosporium cladosporioides* resulting from sequence-based species delimitation analyses. Pathogens.

[6] Sandoval-Denis M., Gené J., Sutton D.A., Wiederhold N., Cano-Lira J.F., Guarro J. (2016). New species of *Cladosporium* associated with human and animal infections. Persoonia.

[7] Schubert K., Groenewald J.Z., Braun U., Dijksterhuis J., Starink M., Hill C.F., Zalar P., de Hoog G.S., Crous P.W. (2007). Biodiversity in the *Cladosporium herbarum* complex (*Davidiellaceae*, *Capnodiales*), with standardization of methods for *Cladosporium* taxonomy and diagnostics. Stud. Mycol..

[8] Bensch K., Braun U., Groenewald J.Z., Crous P.W. (2012). The genus *Cladosporium*. Stud. Mycol..

[9] de Hoog G.S., Guého E., Masclaux F., Gerrits van den Ende A.H., Kwon-Chung K.J., McGinnis M.R. (1995). Nutritional physiology and taxonomy of human-pathogenic *Cladosporium*-*Xylohypha* species. J. Med. Vet. Mycol..

[10] Gugnani H.C., Ramesh V., Sood N., Guarro J., Moin-Ul-Haq, Paliwal-Joshi A., Singh B. (2006). Cutaneous phaeohyphomycosis caused by *Caldosporium oxysporum* and its treatment with potassium iodide. Med. Mycol..

[11] Kong Q.T., Duan Y.Y., Yuan F., Chen J., Liu F., Dang Y.C., Sang H. (2021). Subcutaneous Infection Caused by *Cladosporium sphaerospermum*: A Case Report. Mycopathologia.

[12] Lalueza A., López-Medrano F., del Palacio A., Alhambra A., Alvarez E., Ramos A., Pérez A., Lizasoain M., Meije Y., García-Reyne A. (2011). *Cladosporium macrocarpum* brain abscess after endoscopic ultrasound-guided celiac plexus block. Endoscopy.

[13] Torres-Guerrero E., Arenas R., Hernández-Castro R. (2018). Chromoblastomycosis due to *Cladosporium langeronii*. Molecular diagnosis of an agent previously diagnosed as *Fonsecaea pedrosoi*. An. Bras. Dermatol..

[14] Yang Y.P., Zhong C.J., Yin S.J., Fan Y.M. (2023). Chromoblastomycosis Caused by *Cladosporium tenuissimum* in an Elderly Man. Mycopathologia.

[15] Chew F.L.M., Subrayan V., Chong P.P., Goh M.C., Ng K.P. (2009). *Cladosporium cladosporioides* keratomycosis: A case report. Jpn. J. Ophthalmol..

[16] Giri S., Kindo A.J., Rao S., Kumar A.R. (2013). Unusual causes of fungal rhinosinusitis: A study from a tertiary care centre in South India. Indian J. Med. Microbiol..

[17] Nath R., Barua S., Barman J., Swargiary P., Borgohain M., Saikia L. (2015). Subcutaneous mycosis due to *Cladosporium cladosporioides* and *Bipolaris cynodontis* from Assam, north-east India and review of published literature. Mycopathologia.

[18] Bordoloi P., Nath R., Borgohain M., Huda M.M., Barua S., Dutta D., Saikia L. (2015). Subcutaneous mycoses: An aetiological study of 15 cases in a tertiary care hospital at Dibrugarh, Assam, northeast India. Mycopathologia.

[19] Grava S., Lopes F.A., Cavallazzi R.S., Grassi M.F., Svidzinski T.I. (2016). A rare case of hemorrhagic pneumonia due to *Cladosporium cladosporioides*. J. Bras. Pneumol..

[20] Kumar S., Stecher G., Tamura K. (2016). MEGA7: Molecular Evolutionary Genetics Analysis Version 7.0 for Bigger Datasets. Mol. Biol. Evol..

[21] Ronquist F., Teslenko M., van der Mark P., Ayres D.L., Darling A., Höhna S., Larget B., Liu L., Suchard M.A., Huelsenbeck J.P. (2012). MrBayes 3.2: Efficient Bayesian phylogenetic inference and model choice across a large model space. Syst. Biol..

[22] Korabečná M., Liška V., Fajfrlík K. (2003). Primers *ITS1*, *ITS2* and *ITS4* detect the intraspecies variability in the internal transcribed spacers and 5.8S rRNA gene region in clinical isolates of fungi. Folia Microbiol..

[23] Carbone I., Kohn L.M. (1999). A method for designing primer sets for speciation studies in filamentous ascomycetes. Mycologia.

[24] Bensch K., Groenewald J.Z., Dijksterhuis J., Starink-Willemse M., Andersen B., Summerell B.A., Shin H.-D., Dugan F.M., Schroers H.-J., Braun U. (2010). Species and ecological diversity within the *Cladosporium cladosporioides* complex (*Davidiellaceae*, *Capnodiales*). Stud. Mycol..

[25] Breda L.C.D., Menezes I.G., Paulo L.N.M., de Almeida S.R. (2020). Immune sensing and potential immunotherapeutic approaches to control chromoblastomycosis. J. Fungi..

[26] Torres E., Gil-Beristain J., Lievanos Z., Arenas R. (2010). Chromoblastomycosis associated with a letal squamous cell carcinoma. An. Bras. Dermatol..

[27] Zhou Y.B., Chen P., Sun T.T., Wang X.J., Li D.M. (2016). Acne-Like Subcutaneous Phaeohyphomycosis Caused by *Cladosporium cladosporioides*: A Rare Case Report and Review of Published Literatures. Mycopathologia.

[28] Tritto M., Procop G.W., Billings S.T., Mirkin G., Hao X. (2021). Eumycetoma, A Neglected Tropical Disease in the United States. J. Am. Podiatr. Med. Assoc..

[29] Maubon D., Garnaud C., Ramarozatovo L.S., Fahafahantsoa R.R., Cornet M., Rasamoelina T. (2022). Molecular Diagnosis of Two Major Implantation Mycoses: Chromoblastomycosis and Sporotrichosis. J Fungi..

[30] Iturrieta-González I., García D., Gené J. (2021). Novel species of *Cladosporium* from environmental sources in Spain. MycoKeys.

[31] Becker P.T., de Bel A., Martiny D., Ranque S., Piarroux R., Cassagne C., Detandt M., Hendrickx M. (2014). Identification of filamentous fungi isolates by MALDI-TOF mass spectrometry: Clinical evaluation of an extended reference spectra library. Med. Mycol..

[32] Tóth R., Nosek J., Mora-Montes H.M., Gabaldon T., Bliss J.M., Nosanchuk J.D., Turner S.A., Butler G., Vágvölgyi C., Gácser A. (2019). *Candida parapsilosis*: From Genes to the Bedside. Clin. Microbiol. Rev..

[33] van Asbeck E.C., Clemons K.V., Stevens D.A. (2009). *Candida parapsilosis*: A review of its epidemiology, pathogenesis, clinical aspects, typing and antimicrobial susceptibility. Crit. Rev. Microbiol..

[34] Govrins M., Lass-Flörl C. (2023). *Candida parapsilosis* complex in the clinical setting. Nat. Rev. Microbiol..

[35] Pfaller M.A., Carvalhaes C.G., DeVries S., Rhomberg P.R., Castanheira M. (2022). Impact of COVID-19 on the antifungal susceptibility profiles of isolates collected in a global surveillance program that monitors invasive fungal infections. Med. Mycol..

[36] Díaz-García J., Gómez A., Alcalá L., Reigadas E., Sánchez-Carrillo C., Pérez-Ayala A., de la Pedrosa E.G.-G., González-Romo F., Merino-Amador P., Cuétara M.S. (2022). Evidence of Fluconazole-Resistant *Candida parapsilosis* Genotypes Spreading across Hospitals Located in Madrid, Spain and Harboring the Y132F ERG11p Substitution. Antimicrob. Agents Chemother..

[37] Queiroz-Telles F., Fahal A.H., Falci D.R., Caceres D.H., Chiller T., Pasqualotto A.C. (2017). Neglected endemic mycoses. Lancet Infect. Dis..

[38] Santos D.W.C.L., de Azevedo C.M.P.E.S., Vicente V.A., Queiroz-Telles F., Rodrigues A.M., de Hoog G.S., Denning D.W., Colombo A.L. (2021). The global burden of chromoblastomycosis. PLoS Negl. Trop. Dis..

[39] Queiroz-Telles F., Esterre P., Perez-Blanco M., Vitale R.G., Salgado C.G., Bonifaz A. (2009). Chromoblastomycosis: An overview of clinical manifestations, diagnosis and treatment. Med. Mycol..

[40] Bonifaz A., Paredes-Solís V., Saúl A. (2004). Treating chromoblastomycosis with systemic antifungals. Expert. Opin. Pharmacother..

[41] Bassas-Vila J., Fuente M.J., Guinovart R., Ferrándiz C. (2014). Cromomicosis. Respuesta al tratamiento con crioterapia y terbinafina. Actas Dermosifiliogr..

[42] Langner S., Staber P.B., Neumeister P. (2008). Posaconazole in the management of refractory invasive fungal infections. Ther. Clin. Risk Manag..

